# A Study of Thin Films Based on Polylactide and Vanillic Acid-Crucial Properties Relevant to Packaging

**DOI:** 10.3390/polym17070882

**Published:** 2025-03-26

**Authors:** Ewa Olewnik-Kruszkowska, Magdalena Wrona, Anna Rudawska

**Affiliations:** 1 Chair of Physical Chemistry and Physicochemistry of Polymers, Faculty of Chemistry, Nicolaus Copernicus University in Toruń, Gagarin 7 Street, 87-100 Toruń, Poland; 2 Institute of Bio- and Geosciences 2, Forschungszentrum Jülich GmbH, 52428 Jülich, Germany; m.wrona@fz-juelich.de; 3 Faculty of Mechanical Engineering, Lublin University of Technology, Nadbystrzycka 36 St., 20-618 Lublin, Poland; a.rudawska@pollub.pl

**Keywords:** polymeric films, polylactide, vanillic acid, packaging materials, storage, antioxidative properties

## Abstract

In recent years, there has been a growing emphasis on packaging made from biodegradable materials. These materials not only help to reduce environmental impact, but also extend the shelf life of food products, thereby contributing to a significant reduction in food waste. In order to develop novel biodegradable polymeric films for use as active packaging, polylactide materials filled with vanillic acid were prepared. Analyses were conducted to determine the influence of vanillic acid on the structural morphology and key food storage properties of the resulting films, including water vapor resistance, mechanical properties, UV barrier properties, opacity, and antioxidant activity. The effect of the vanillic acid on the firmness of blueberries packed in films with and without the additive was evaluated. The research demonstrated that incorporating vanillic acid into polylactide significantly enhanced the UV barrier properties of the resulting materials. Furthermore, the resulting antioxidant activity contributed to extending the freshness of the stored blueberries. The addition of vanillic acid did not substantially affect the transparency of the films, maintaining the visibility of the fruit.

## 1. Introduction

In recent years, biodegradable polymer materials have garnered significant interest, due to the increasing emphasis on sustainability and the demand for eco-friendly and natural solutions in the packaging industry. Traditional plastics, widely used in product packaging, pose a serious environmental challenge, due to their prolonged decomposition and negative impact on the environment. A promising biodegradable polymer that is gaining increasing recognition is polylactide (PLA). Polylactide is characterized by biocompatibility and good mechanical properties, making it an attractive material for packaging production. However, due to its low resistance to microorganisms, it requires modification to meet the demands of active packaging [1,2]. The extension of shelf life can be achieved by incorporating antibacterial substances or antioxidants into the packaging, which are compounds that are able to hinder the negative effects of free radicals [3]. In an effort to improve the antioxidative properties of the formed packaging materials, the following types of molecules have been studied and presented in the literature. The main groups of compounds with antioxidative properties include lignans, stilbenes, flavonoids, phenylpropanoids, terpenoids, alkaloids, and phenolic compounds in the form of acids and their derivatives [4].

Polylactide, which serves as the polymer matrix studied in this work, has been modified by many researchers using various substances that enhance its antioxidative properties, provide UV resistance, and improve its antibacterial properties. A significant group of polylactide-based materials comprises films containing various essential oils. Several studies have been conducted on polylactide with the addition of cinnamon oil [5,6,7], whose presence in packaging significantly improved the resistance of stored poultry produce to bacteria such as *Listeria monocytogenes* and *Salmonella typhimurium*. In the work of Ardjoum et al. [8], *Thymus vulgaris* essential oil and an ethanolic extract of *Mediterranean propolis* were used. The authors proved that applying a combination of the two components results in obtaining promising antibacterial films based on polylactide. Ahmed et al. [9] investigated zinc oxide (ZnO) nanoparticles and clove essential oil incorporated into a polylactide-poly(ε-caprolactone) polymeric matrix. The obtained materials were characterized as antibacterial films effective against *Staphylococcus aureus* and *Escherichia coli*. Similarly, in the work of Olewnik-Kruszkowska et al. [10], a comparison of the crucial properties of materials based on polylactide and selected essential oils—including tea tree, clove, rosemary, and grapefruit oils—was conducted. The study established that all active ingredients significantly influenced the thermal and mechanical properties of the materials. Moreover, the PLA-based film containing clove oil exhibited strong antioxidative properties. Nasution et al. [11] presented a comprehensive review of antibacterial and antioxidative agents derived from natural extracts incorporated into polylactide, along with their impact on the physico-mechanical and antibacterial properties of the obtained materials and the shelf life of perishable foods. The study confirmed that polylactide modified with active compounds is a key element in developing the innovative packaging sector. Among the numerous active compounds introduced into the polylactide matrix and thoroughly examined by researchers are berberine [12], quercetin [13], and cardanol amine-functionalized graphene [14], as well as phenolic compounds such as cinnamic acid and ferulic acid [15,16]. It should be stressed that all the applied modifications significantly affect the shelf life and quality of food.

In this study, vanillic acid (VA)—a phenolic acid—was used as an active ingredient. Phenolic acids, including caffeic acid and ferulic acid, are present in many plant-based products, such as cereal grains, coffee, vegetables, and fruit. They exhibit strong antioxidative properties, which help protect against oxidative damage [17,18].

Vanillic acid (4-hydroxy-3-methylbenzoic acid) is a natural derivative of benzoic acid. It is found primarily in vanilla seeds, but also in blueberries, strawberries, nuts, potatoes, and cereals [19,20]. Due to its properties, vanillic acid has recently attracted the interest of researchers. In their study, Eelager et al. [21] analyzed the mechanical, antimicrobial, and antioxidative properties of active films based on chitosan and poly (vinyl alcohol) with the addition of vanillic acid, designed to extend the shelf life of green chili. The impact of incorporating vanillic acid and five other phenolic compounds into chitosan was investigated by Liu et al. [22]. Meanwhile, in their study, Bakar et al. [23] examined chitosan-based materials grafted with vanillic acid. In the case of the indicated works, the obtained results were satisfactory, particularly in terms of UV barrier properties and antibacterial activity. For this reason, in the present work, as mentioned above, phenolic acid (in the form of vanillic acid) was incorporated into a polylactide-poly(ethylene glycol) system. The structural and thermal properties of the obtained materials were evaluated. In addition, features crucial in terms of food packaging, such as antioxidant and mechanical properties, water vapor permeability, transparency, and UV barrier resistance, were analyzed. It has to be noted that modification of the aforementioned properties significantly affects the shelf life and quality of the stored blueberries.

## 2. Materials and Methods

### 2.1. Materials

Polylactide with an average molecular weight of 155,500 Da was delivered by Nature Works^®^ (Minnetonka, MN, USA). Poly(ethylene glycol) with M_w_ = 1500, DPPH, and vanillic acid were provided by Sigma-Aldrich (Steinheim, Germany). Chloroform, acetone, and calcium chloride were purchased from Avantor Performance Materials Poland S.A. (Gliwice, Poland). Blueberries of the Brightwell variety, originating from Chile, were used during the storage test.

### 2.2. Formation of Polylactide-Based Films

The PLA-based composites filled with vanillic acid were obtained using the casting method. Dried polylactide was dissolved in chloroform (3% *m*/*v*), then poly(ethylene glycol) was introduced as a plasticizer (5% *w*/*w*, PLA). In the next step, vanillic acid (1, 2, and 3% *w*/*w* of PLA) dissolved in 5 mL of acetone was added to the PLA-PEG solution (50 mL). After 1 h of mixing, the solutions were poured out onto Petri dishes and stored at 20 °C for 48 h. Particular samples were identified as follows: P—PLA-PEG system, V—vanillic acid; 1, 2, 3 indicate the percentage of vanillic acid. The thickness of the studied materials was analyzed by means of an Absolute Digimatic Indicator (Sylvac S229, Swiss, Yverdon, Switzerland). It was established that the thickness does not significantly depend on the vanillic acid amount. In Table 1, the real photos of the studied materials, as well as the values of thickness, are shown.

### 2.3. Methods of Analysis

#### 2.3.1. Fourier Transform Infrared Analysis

The structure of the formed films with and without the addition of vanillic acid was studied by means of a Nicolet iS10 (Thermo Fisher Scientific, Waltham, MA, USA). The parameters of 64 scans, a resolution of 4 cm^−1^, and a frequency range of 600–4000 cm^−1^ were applied during the recording of all spectra.

#### 2.3.2. SEM and AFM Study

The morphology of the obtained PLA-based films was analyzed by means of a scanning electron microscope Quanta 3D FEG (FEI Company, Hillsboro, OR, USA). The topography of materials with and without vanillic acid was studied by means of an atomic force microscope with a scanning SPM probe of the NanoScope MultiMode type (Veeco Metrology, Inc., Santa Barbara, CA, USA). The Nanoscope IIIa software (Veeco Metrology, Inc., Santa Barbara, CA, USA) was applied to determine the roughness parameters, such as the root mean square (R_q_) and arithmetical mean deviation (R_a_).

#### 2.3.3. DSC Analysis

Differential scanning calorimetry was used to evaluate the temperatures of glass transition (T_g_), cold crystallization (T_c_), and melting (T_m_), and to calculate the values of the degree of crystallinity (X_c_), cold crystallization enthalpy (ΔH_c_), and melting enthalpy (ΔH_m_). All analyses were performed using the thermoanalyzer manufactured by Polymer Laboratories (Epsom, UK), in a nitrogen atmosphere, with a heating rate of 10 °C/min, and within a temperature range between 25 and 200 °C.

#### 2.3.4. Mechanical Properties

Changes in the mechanical properties, such as Young’s modulus (E), elongation at break (ε), and tensile strength (σ_m_), of the obtained films were evaluated using an EZ-SX machine (Shimadzu, Kyoto, Japan). The crosshead speed was 10 mm·min^−1^, with an applied force of 100 N.

#### 2.3.5. Assessment of Antioxidative Properties

The antioxidative activity of the obtained films filled with vanillic acid was analyzed by means of the DPPH method. The analyses were performed according to the methodology described in our previous work [10]. The obtained extracts, after shaking 0.6 g of films in 6 g of methanol, were introduced in different volumes into the DPPH solution (30 µg/g) and stored in the dark for 30 min. In the next stage, the absorbance of the stored solutions was measured at 515 nm using a Halo DB-20 spectrophotometer (Dynamica Scientific Ltd., Newport Pagnell, UK). The obtained results allowed for the calculation of the volume providing 50% inhibition (IC_50_). All the measurements were performed in triplicate.

#### 2.3.6. Water Vapor Transmission Rate

The effect of vanillic acid on the water vapor transmission rate was studied in accordance with the methodology described in our previous work [10]. During the analysis, the changes in the CaCl_2_ weight were checked every 24 h within a period of one week. It should be noted that the relative humidity was 75%, and the temperature during storage was 30 °C. The WVTR was calculated using the following Equation (1):

(1) $$WVTR=\frac{rate\;of\;moisture\;absorption\;by\;dessicant}{surface\;area\;of\;the\;specimen}\;\left[\frac{g}{m^{2}\times h}\right]$$

#### 2.3.7. Transparency and UV Barrier Properties

To evaluate UV protection, the optical properties of all examined materials were analyzed. Spectra were recorded within the 200–800 nm range using a Halo DB-20 spectrophotometer (Dynamica Scientific Ltd., Newport Pagnell, UK). Each sample underwent triplicate analysis. The UV-blocking effectiveness was assessed in both the *UVA* and *UVB* ranges, utilizing Equations (2) and (3) for this purpose:

(2) $$UVA_{blocking}=100-T_{UVA}$$

(3) $$UVB_{blocking}=100-T_{UVB}$$

The measurement of absorbance at 600 nm (*A*_600_) allowed for the establishment of the transparency (*T*) of the studied materials, according to Equation (4):

(4) $$T=\frac{A_{600}}{d}\;\left[{mm}^{-1}\right]$$

#### 2.3.8. Blueberry Storage and Firmness

Fresh blueberries were carefully selected and covered with the obtained films. The formed packages were stored for one week in the same conditions as used for the original samples during the water vapor transmission rate study (relative humidity—75%, temperature during storage—30 °C). The changes in the weight of blueberries were checked every 24 h, while the firmness of individual berries was tested before and at the end of the storage period. The firmness of blueberries was tested using the EZ-SX machine (Shimadzu, Kyoto, Japan). A cylindrical probe (20 mm in diameter) was employed to examine the blueberries’ response to compression, measuring the force required to achieve 20% deformation of the fruit.

#### 2.3.9. Statistics

Student’s *t*-test was conducted to determine whether the differences between films with varying concentrations of vanillic acid were statistically significant (*p* ≤ 0.05). The null hypothesis assumed no difference between the samples. If the computed *t*-value was greater than the critical *t*-value from the statistical table, the samples were deemed significantly different, leading to rejection of the null hypothesis

## 3. Results and Discussion

### 3.1. Structural Study by Means of FTIR Technique

**Figure 1:**
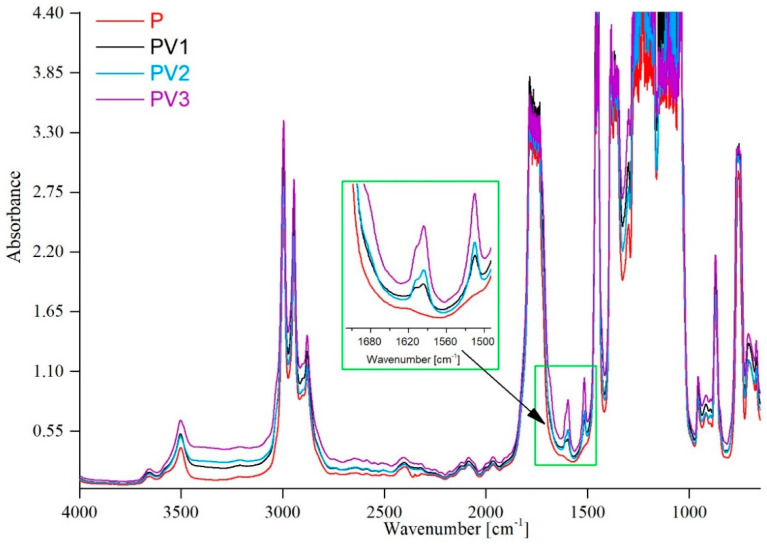
Spectra of PLA-based materials without and with incorporation of vanillic acid.

The Fourier Transform Infrared analysis allowed us to answer the question of whether the addition of vanillic acid can influence the structure of materials based on polylactide. Figure 1 presents the spectra of films with varying amounts of vanillic acid, as well as of those without any addition. The structure of materials composed of polylactide and poly (ethylene glycol) has been described in detail in our previous work [10,12,13]. In the present study, the focus was on the changes induced by the introduction of vanillic acid into the system in various amounts. The position of the bands of individual atomic groups present in the structure of vanillic acid was described in detail by Gonzalez-Baró [24]. To describe the particular bands, the following acronyms were used: ν—stretching vibrations, δ—in-plane deformation vibrations. According to the available literature, changes in the spectra caused by the addition of vanillic acid are visible in the regions of approx. 3484 cm^−1^ and 3091 cm^−1^, corresponding to νOH (phenol), νOH (COOH), and νCH (ring) vibrations, respectively. Particularly noticeable are the bands ascribed to νC=O and δOH (COOH) vibrations at 1683 cm^−1^, as well as those in the enlarged area in Figure 1, namely at 1580 cm^−1^, corresponding to the following vibrations: ν (ring), δOH (all), δCH (all), and, at 1522 cm^−1^, relating to ν (ring) and δCH (all). Moreover, a very strong vibration that can be ascribed to the ν(C–O) group present in the carboxylic acid has been recorded at 1300 cm^−1^. Similar observations regarding the bands belonging to vanillic acid were described by Hong et al. [25].

Moreover, bands indicative of the polymeric matrix, consisting of PLA and PEG, were assigned. The bands at 3656 cm^−1^ and 3500 cm^−1^ clearly indicate the presence of -OH groups at the ends of the PLA and PEG chains. High and broad bands at 2995 cm^−1^ and 2945 cm^−1^ belong to the vibrations of the -CH_3_ group present in PLA, while -CH_2_ group vibrations observed at 2877 cm^−1^ represent PEG. Additionally, the absorption band at 1765 cm^−1^ relates to the carbonyl group (-C=O) of polylactide, while the bands recorded at 1206 cm^−1^ and 1126 cm^−1^ correspond to stretching vibrations of -C-O-C [13].

Particular attention should be paid to the broad band at 3100–3500 cm^−1^, which may indicate the formation of hydrogen bonds in the studied systems. In the case of polylactide, the structure contains carbonyl (C=O) groups in the polymer chain, which can act as hydrogen bond acceptors. Vanillic acid, on the other hand, contains hydroxyl (-OH) and carboxyl (-COOH) groups, which can act as hydrogen bond donors. Additionally, the carbonyl group in the carboxylic acid can also serve as a hydrogen bond acceptor. Such a system provides numerous possibilities for hydrogen bond formation. Namely, the hydroxyl (-OH) groups in vanillic acid can form hydrogen bonds with the carbonyl groups present in polylactide. Additionally, the carbonyl groups of vanillic acid can also participate in hydrogen bond formation with other donors, such as the terminal hydroxyl groups of polylactide or poly (ethylene glycol). The formation of hydrogen bonds between the polymer matrix and vanillic acid was observed in the work of Eelager et al. [21].

### 3.2. Morphology and Topography Evaluation

**Figure 2:**
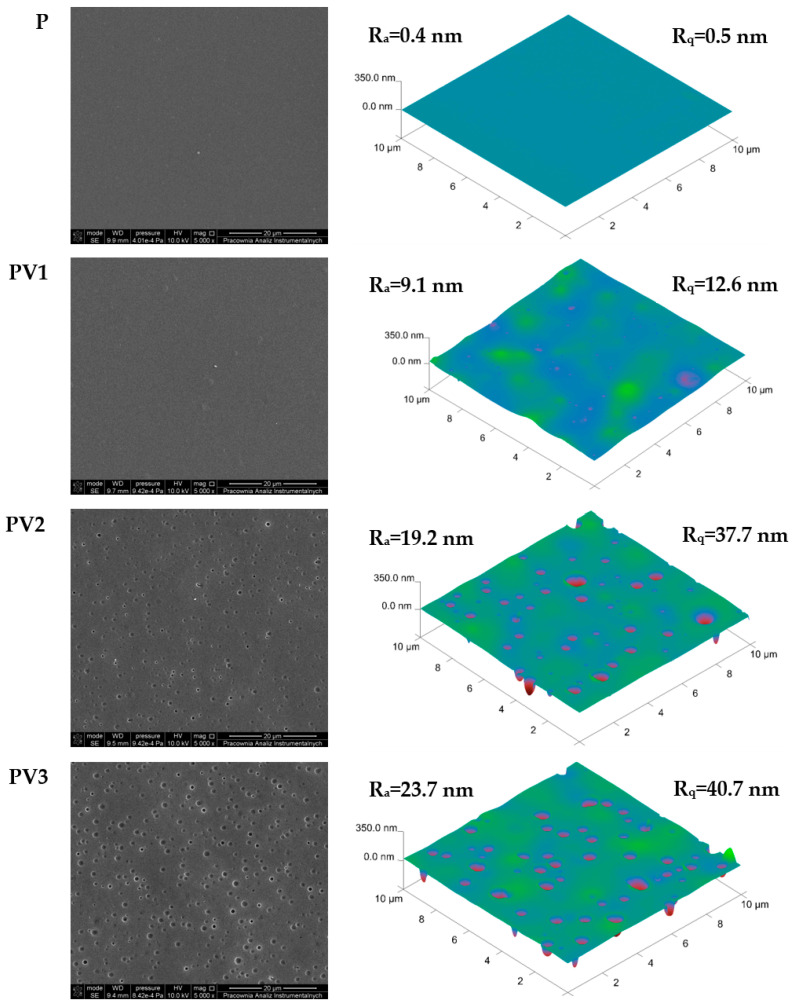
SEM and AFM pictures of the studied materials.

In Figure 2, data obtained by means of scanning electron microscopy and atomic force microscopy are shown. It is well known that most polymeric materials modified with various types of additives change morphology and topography [12,26,27,28,29].

While the surface of the control sample is smooth, without any irregularities or cracks, the introduction of vanillic acid into the polymer matrix leads to the formation of numerous indentations on the surface of the resulting films. The following correlation can be observed: the higher the vanillic acid content in the system, the more indentations appear on the surface of the polylactide-based film. This simultaneously causes an increase in roughness parameters such as the root mean square (R_q_) and arithmetical mean deviation (R_a_). It should be noted, however, that the increase in roughness parameters is not as significant as in the case of polylactide modification with quercetin [13], piper betel leaf ethanolic extract [30], antibacterial agent based on zwitterionic [31], or polylactide-polycaprolactone with zinc oxide and clove essential oil incorporated [32]. In the case of the mentioned additives, the indentations form on the surface of the PLA-based materials due to the drying process, microphase separation, and differences in the evaporation rates of the solvent mixtures used [10].

However, it should be noted that there are more factors contributing to the formation of these changes [33,34,35,36,37]. Most commonly, indentations on the surface result from interphase incompatibility between components, leading to the formation of microphases and phase separation, which, in turn, causes the development of pores or holes. Another possible reason is the volatility of the additives introduced into the system. When they evaporate during film formation, they cause voids to remain within the material’s structure. Additionally, differences in surface tension can also contribute to the formation of indentations, as some additives may affect wettability and surface tension, leading to a heterogeneous film structure and the occurrence of defects.

In the case of the studied polylactide-based films with the addition of vanillic acid, apart from potential phase separation, the possible role of hydrogen bonds in the formation of indentations should be considered. It should be noted that hydrogen bonds may alter the kinetics of solvent evaporation, leading to local differences in surface tension and the Marangoni effect, ultimately resulting in uneven material retraction [38,39].

### 3.3. Determination of Thermal Properties of PLA-Based Materials

**Figure 3:**
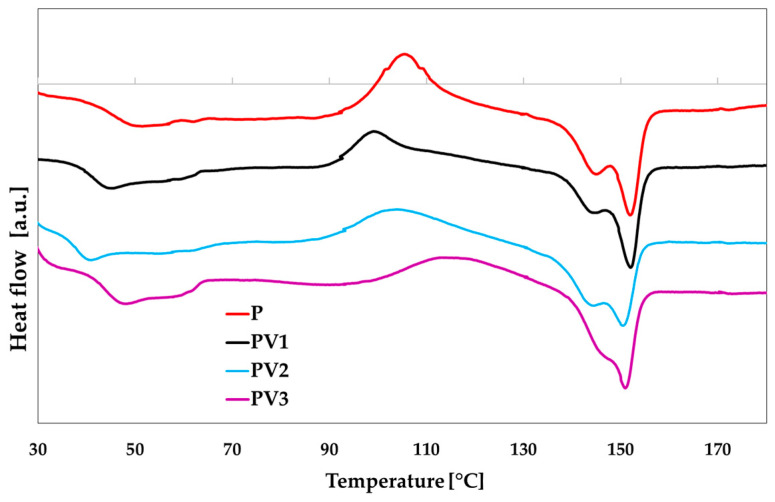
DSC curves of the studied PLA-based materials.

Changes in the thermal properties of the formulated system depend on the type of polymer, the introduced additive, and its quantity. In Figure 3, the thermograms of all discussed materials are shown. Table 2 presents the values of thermal parameters, such as the temperatures of melting (T_m_), cold crystallization (T_c_), and glass transition (T_g_), as well as the melting enthalpy (ΔH_m_) and cold crystallization enthalpy (ΔH_c_). The values of the degree of crystallinity (X_c_) were also calculated.

Based on the obtained data, it can be observed that the introduction of vanillic acid into the PLA-PEG system causes a decrease in the glass transition temperature. A similar effect was noted in the study by Ordonez et al. [15], where cinnamic acid was added to polylactide. However, in the case of the temperature of cold crystallization, the addition of vanillic acid in amounts of 1% and 2% results in a decrease in T_c_, although the peak itself becomes broader and more diffused. It can therefore be assumed that the active component may interact with the PLA chains through hydrogen bonding, thereby altering their arrangement, which allows them to form ordered structures at a lower temperature. At the same time, it is observed that there are fewer ordered structures, as evidenced by the reduced ΔH_c_ and ΔH_m_ values. Interestingly, the introduction of 3% vanillic acid into the system, despite lowering both the crystallization and melting enthalpies, simultaneously causes an increase in the cold crystallization temperature. It can be assumed that an increase in the amount of the active component causes the distances between the polylactide chains to expand to such an extent that the crystallization process becomes significantly hindered, leading to a rise in T_c_. It should also be noted that with increasing additive content, it becomes increasingly difficult to observe two melting peaks, which indicate the formation of two crystalline forms of polylactide [40]. Polylactide (PLA) can crystallize into two main crystalline forms: α (alpha) and β (beta), depending on processing conditions, introduced additives, and polymer chain orientation. The α form is more stable, and is characterized by an orderly arrangement of polymer chains. The β form is less stable and has a less ordered structure. Vanillic acid, which acts as a plasticizer in the studied systems, disrupts the crystalline structure, but does not cause a significant change in the melting temperature range. However, instead of two distinct melting peaks, only one peak, corresponding to the α form, is clearly visible. The literature suggests that additives may induce a loosening of the PLA structure, favoring the β form, which is more flexible and less stable. However, it should be noted that an excess of plasticizer may lead to amorphization [41,42,43,44,45,46].

### 3.4. Changes in Mechanical Properties

**Figure 4:**
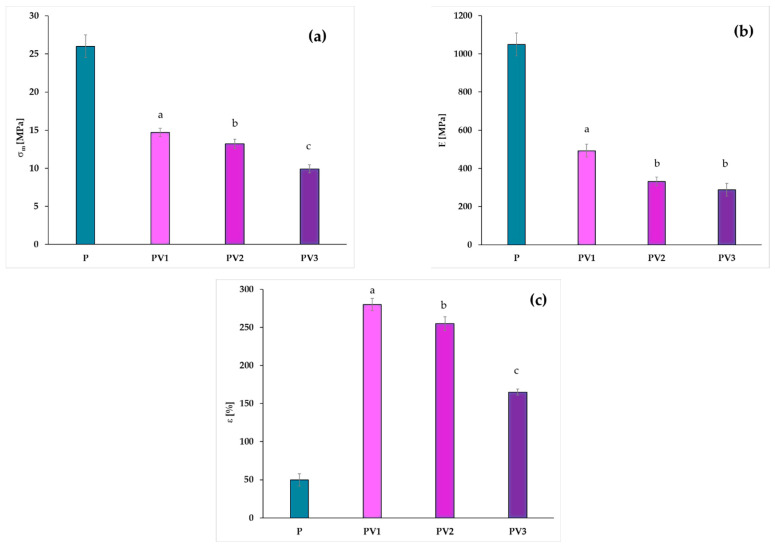
Differences in mechanical properties of studied materials: (**a**) tensile strength (σ_m_), (**b**) Young’s modulus (E), (**c**) elongation at break (ε) (different letters (a–c) indicate significative (*p* ≤ 0.05) differences between samples).

The introduction of low-molecular-weight compounds into the polymer matrix affects the mechanical properties of the final product in different ways, depending on the interactions between the components of the system. In the case of food packaging, mechanical properties are one of the key parameters that can determine the potential applications of a given material. The introduced additives can interact with the polymer matrix through hydrogen bonding and crosslinking processes, acting as nucleating agents of crystallization or as plasticizers [26,27,28,29,47]. In Figure 4, the values of the following mechanical properties for the tested systems are shown: tensile strength (σ_m_), Young’s modulus (E), and elongation at break (ε). The introduction of vanillic acid into the PLA-PEG system results in a decrease in Young’s modulus, which is a parameter that indicates the stiffness of a material in response to tensile stress. In the case of the studied materials, several factors may influence the E value, such as hydrogen bonds, the degree of crystallinity of the materials, and the displacement of polylactide chains caused by the presence of a low-molecular-weight compound in the form of vanillic acid. Hydrogen bonds are considered weak interactions; therefore, the reduction in the Young’s modulus values of the obtained materials is primarily associated with a decrease in the crystallinity of the formed films containing vanillic acid and the plasticizing effect of VA, which reduces intermolecular interactions in polylactide and increases the mobility of PLA polymer chains, as confirmed by the results obtained using the DSC analysis. However, in the case of the PV3 sample, the distance between the polylactide chains caused by the active additive is initially balanced by the presence of hydrogen bonds between the polymer matrix and vanillic acid. It should be noted, however, that the addition of 3% vanillic acid results in a Young’s modulus value which is similar to that of the material containing 2% of the active additive in the polymer matrix. This observation suggests that in the case of sample PV3, the distance between the polylactide chains caused by the active additive is initially balanced by the presence of hydrogen bonds between the polymer matrix and vanillic acid.

During the analysis of the elongation at break, it was observed that the addition of 1% vanillic acid significantly increased the values of this parameter. Although the values remained significantly higher than those of the control sample, introducing higher amounts of vanillic acid led to a gradual decrease in this parameter. This, in turn, indicates that vanillic acid can be an excellent plasticizer within a specific concentration range. The incorporation of more significant amounts of this compound resulted in a reduction in elongation at break. This may suggest limited miscibility of the components, as well as a reduction in interactions between polylactide chains, which simultaneously contributes to a decrease in tensile strength.

Because the degree of crystallinity significantly affects the stiffness and strength of the materials, the decrease noted when comparing the film containing vanillic acid with the control sample without an active compound is of primary relevance [48,49].

A similar effect of a reduction in Young’s modulus values and tensile strength was observed by Ordonez et al. [15,50] in a study where ferulic acid and cinnamic acid were introduced into polylactide. However, in the case of these materials, the incorporation of active additives also led to a decrease in elongation at break, which was attributed to the lack of a plasticizing effect of the studied acids, in contrast to vanillic acid. The available literature indicates that vanillic acid has been introduced as an active component into chitosan [22]. The obtained results showed that the addition of vanillic acid weakened the intramolecular interactions within chitosan and disrupted the formation of its crystalline structure, leading to a reduction in all mechanical properties. Different results were reported in the study by Eelager. et al. [21], where materials based on chitosan and poly (vinyl alcohol) with the addition of vanillic acid were analyzed. In this study, it was observed that materials containing vanillic acid exhibited significantly higher Young’s modulus values and tensile strength, while at the same time, elongation at break was inversely proportional to the content of the active component. The observed effects were explained by the formation of intermolecular hydrogen bonds and Schiff’s bases.

In summary, based on the obtained results, it can be concluded that vanillic acid may be a promising additive to the polylactide matrix, significantly improving the flexibility of the resulting packaging materials.

### 3.5. Examination of WVTR

**Figure 5:**
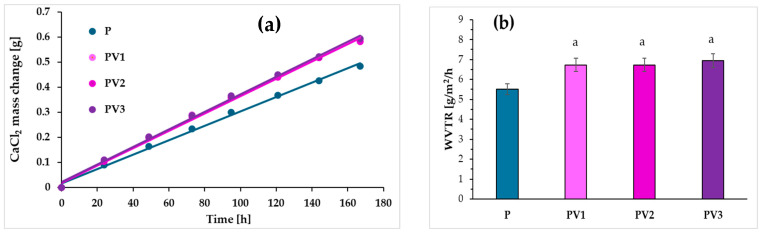
(**a**) Time vs. CaCl_2_ weight changes; (**b**) differences in water vapor transmission rate of studied materials (letter a indicate significative (*p* ≤ 0.05) differences between samples).

The water vapor transmission rate (WVTR) is a crucial parameter considered in the production of packaging for both medical and food products. This study examined the effect that the addition of vanillic acid has on the water vapor transmission rate in PLA-based films with PEG. To determine the WVTR values, changes in the mass of CaCl_2_ over time were analyzed for PLA-based materials with and without the addition of poly (ethylene glycol) and vanillic acid (Figure 5a). Based on the obtained results, it can be observed that for all materials, the mass of CaCl_2_ increased over time. Figure 5b presents the water vapor transmission values for the tested materials. The obtained data allowed us to determine that the addition of vanillic acid led to an increase in WVTR compared to the control sample, in the case of which the water vapor transmission rate was approximately 5.5 (g m^−2^ h^−1^). It can therefore be deduced that the water vapor transmission rate changed after the addition of vanillic acid due to its impact on the polymer structure. The addition of vanillic acid reduces the degree of crystallinity, resulting in an increased accumulation of amorphous regions in the polymer, which, in turn, enhances water vapor transmission. Moreover, the low-molecular-weight additive increases the distances between polymer chains, which also affects the WVTR value. At the same time, it should be noted that increasing the content of vanillic acid to 2% and 3% in the PLA-PEG system did not lead to a significant increase in water vapor permeability. Regardless of the amount of the acid, the WVTR value increased to approximately 7 (g m^−2^ h^−1^). This may indicate a balance between the proportion of the amorphous phase, the increase in the amount of the low-molecular-weight compound, and the formation of hydrogen bonds.

Conversely, in the work of Eelager et al. [21], where chitosan- and poly (vinyl alcohol)-based films with the addition of vanillic acid were examined, it was found that the active component introduced into the system caused a slight decrease in the WVTR values of the obtained materials. The changes in WVTR were explained by the presence of covalent bonds between vanillic acid and chitosan, which led to a reduction in the number of hydroxyl and amine hydrophilic groups in chitosan. The same tendency was observed by Bakar et al. [23], where materials based on chitosan and vanillic acid were analyzed. However, it is difficult to compare the properties of chitosan-based materials with the studied systems, as it should be noted that polylactide does not form covalent bonds with vanillic acid. In studies by Ordonez et al. [15,50], it was also found that the addition of both ferulic acid and cinnamic acid reduced the water vapor permeability of PLA-based films. However, it is important to consider the structure of phenolic acids. Vanillic acid contains a hydroxyl group (-OH) and a methoxy group (-OCH_3_), which enhance its potential to form hydrogen bonds with water. In contrast, cinnamic acid only incorporates a carboxyl group (-COOH) and a double bond in the side chain, making it less hydrophilic than vanillic acid. Ferulic acid, on the other hand, contains both a hydroxyl group (-OH) and a methoxy group (-OCH_3_), similarly to vanillic acid, but also features a longer, less polar segment associated with the double bond. This explains why cinnamic acid and ferulic acid improve the water vapor resistance of PLA-based materials, whereas vanillic acid leads to a slight, yet noticeable, increase in the permeability of polylactide films.

### 3.6. Differences in Antioxidative Properties

**Figure 6:**
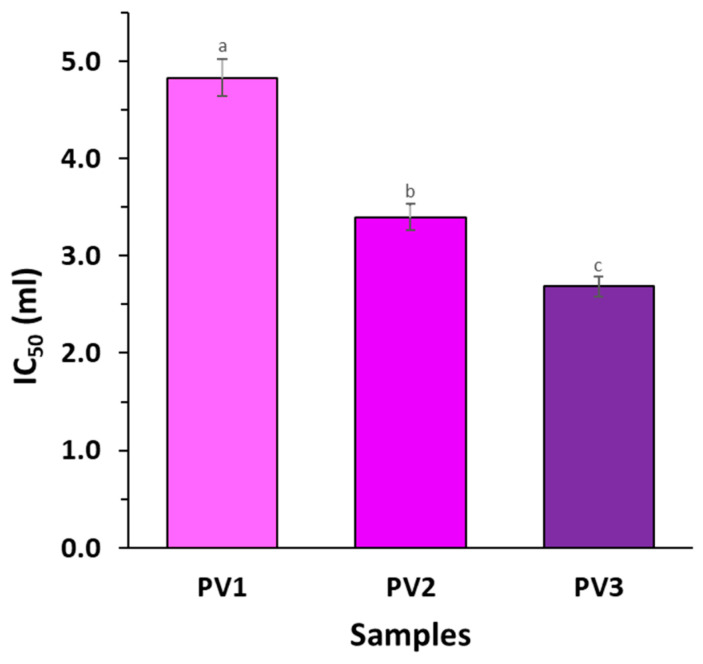
Antioxidant activity of studied materials filled with vanillic acid (different letters (a–c) indicate significative (*p* ≤ 0.05) differences between samples).

The antioxidant capacity (CAOX) of the PLA films with vanillic acid was evaluated through the implementation of a DPPH radical scavenging assay (Figure 6). Furthermore, a statistical evaluation of the antioxidant activity outcomes was conducted using Student’s test. In the case of the DPPH assay, a lower DPPH radical scavenging value corresponded to a stronger antioxidant capacity. This inverse relationship means that samples with lower absorbance or percentage inhibition values exhibited greater efficiency in neutralizing free radicals. Consequently, materials or compounds demonstrating the lowest DPPH values possessed the highest antioxidant potential.

The results indicate that increasing the concentration of vanillic acid in the polylactide-based polymer increases its antioxidant activity. In the case of the most concentrated sample (PV3), a lower volume of active polymer extract was required to reduce the concentration of DPPH radical by half. Therefore, the highest antioxidant capacity was obtained for polymer PV3 with 3% active ingredient, while the lowest CAOX was obtained for sample PV1 with a concentration of 1% vanillic acid. Sample PV3 had a 50% higher CAOX than sample PV1. All the samples were significantly (*p* < 0.05) different.

This trend is consistent with previous studies in the literature, which have shown that phenolic compounds, such as vanillic acid, contribute significantly to antioxidant capacity, due to their ability to donate hydrogen atoms and neutralize free radicals.

For instance, research on vanillic acid incorporated into biopolymeric matrices, such as chitosan and poly (vinyl alcohol)-based films, has shown a similar dose-dependent increase in antioxidant properties (measured by DPPH) [21]. What reinforces the idea that antioxidant performance improves with increased active compound loading? Moreover, studies on phenolic acid-functionalized polymers, using active compounds such as ferulic acid, vanillic acid, gallic acid, and salicylic acid, have also reported enhanced radical scavenging efficiency with the addition of phenolic acid [22]. Also, a study on the addition of vanillic acid to hydrogel films showed that this active agent notably enhanced the scavenging ratios of DPPH [23].

### 3.7. Evaluation of Transparency and UV Barrier

**Figure 7:**
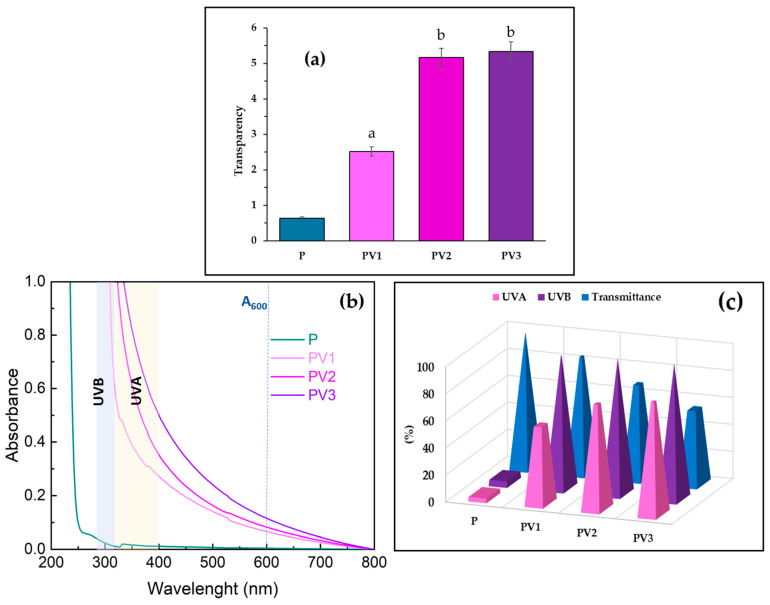
(**a**) Transparency; (**b**) UV spectra of studied materials; (**c**) vanillic acid and UV-barrier properties and transmittance at 600 nm (different letters (a, b) indicate significative (*p* ≤ 0.05) differences between samples).

The transparency and *UVA*/*UVB* barrier properties of the obtained PLA-PEG-based materials, both with and without the addition of vanillic acid, were analyzed. Figure 7a presents the transparency results of the tested materials. The transparency of packaging materials is particularly important for consumers, as it affects the visual appeal and esthetics of the packaged product. Transparent packaging allows consumers to see the product in its natural form, which is especially beneficial for fresh fruits and vegetables. Moreover, the opportunity to inspect the product before purchase reduces concerns about its quality and freshness, contributing to increased consumer trust. The introduction of vanillic acid into the PLA-PEG system caused a slight decrease in the transparency of the obtained films. The highest opacity value was observed for the sample containing 3% of the active additive, reaching approximately 5.5. Typically, the addition of components to a polymer matrix leads to reduced transparency. Higher opacity values have been reported after incorporating additives such as quercetin, berberine, essential oils, and deep eutectic solvents [10,12,13,51].

The obtained results confirm that the incorporation of vanillic acid into the PLA-PEG system significantly improved the UV barrier properties of the tested materials. Even the addition of 2% vanillic acid provided nearly 100% protection against *UVB* radiation and 80% protection against *UVA* radiation. This confirms that vanillic acid is a crucial component of packaging materials, and can help prevent the loss of nutritional value, as well as preventing undesirable changes in color and texture.

### 3.8. Storing Blueberries: Firmness and Weight Loss

**Figure 8:**
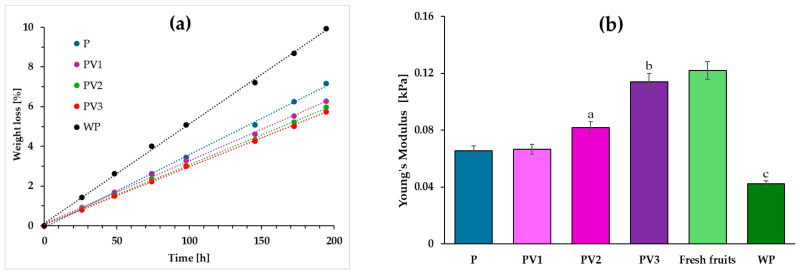
(**a**) Changes in fruit masses; (**b**) Young’s modulus for blueberries before and after storage, (different letters (a–c) indicate significative (*p* ≤ 0.05) differences between samples).

The presence of antioxidant compounds in packaging can significantly improve the shelf life of blueberries, mainly by delaying oxidation processes and slowing down the growth of mold and microorganisms [52,53]. Therefore, the obtained packaging materials, both with and without vanillic acid, were used to test the firmness and weight loss of blueberries over a seven-day storage period. Figure 8a presents the weight changes of blueberries stored without packaging (WP) and those protected by the polymer films. The highest weight loss was observed in unpackaged fruits (approximately 10%), and in the case of blueberries stored in films without the active compound (around 7%). In contrast, the presence of vanillic acid reduced weight loss in the stored fruits. The same trend was observed in the Young’s modulus of the tested fruits (Figure 8b). To determine the effect of vanillic acid on the firmness of the tested fruits, the letters shown in Figure 8b indicate significant differences in the Young’s modulus values recorded for blueberries packed in polymeric film without the incorporation of vanillic acid, and for blueberries in packages with added VA, as well as for fruit stored without packaging. The Young’s modulus values relating to fresh fruit before storage were not taken into account in the statistical analysis.

Weight loss in blueberries is inherently linked to a decrease in firmness; therefore, the lower the weight loss, the longer the blueberries retain their firmness, ultimately extending their shelf life. Antioxidant packaging can protect fruit from weight loss primarily by impeding oxidation processes, which delays aging and dehydration. Slower oxidation results in less structural degradation of the fruit, limiting shrinkage and water loss. Moreover, vanillic acid, as an antioxidant compound, can inhibit the growth of mold and bacteria, which accelerate fruit spoilage and contribute to greater weight loss. Through these mechanisms, antioxidant packaging helps to maintain the freshness, firmness, and weight of fruits for a longer period. The extension of blueberry freshness due to the incorporation of an active compound into the polymer matrix has also been observed by other researchers. Compounds such as quercetin, lemon essential oil, grapefruit seed extract, propolis, and nano-carriers of salicylate have been shown to reduce the loss of firmness in blueberries [13,54,55,56]. The addition of these antioxidant compounds to packaging has extended the shelf life of blueberries, improved their quality, and reduced losses, which is particularly important for transportation and storage.

## 4. Conclusions

This study analyzed the effect of vanillic acid on the structure and crucial physicochemical properties of PLA-based materials with the addition of poly (ethylene glycol). The obtained results confirmed that incorporating vanillic acid into the PLA-PEG polymer matrix leads to the formation of hydrogen bonds between the polymer components and the active compound. These hydrogen bonds allow vanillic acid to act as a plasticizer in the developed systems, while only slightly reducing water vapor permeability.

Furthermore, it should be emphasized that the obtained materials provide an excellent barrier against *UVA* and *UVB* radiation, which is one of the key factors determining the shelf life of food products. Importantly, the films containing vanillic acid exhibited antioxidant properties, contributing to the extended freshness and firmness of stored blueberries.

In summary, vanillic acid, a non-toxic compound naturally occurring as a metabolite of vanillin in the human body and certain plants, represents an extremely promising component for engineering novel, active packaging materials. The developed polylactide-based materials with vanillic acid are characterized by flexibility, UV barrier properties, and the potential to extend the shelf life of consumable staples, making them promising active packaging materials.

## Figures and Tables

**Table 1:** Photos and values of thickness of studied materials.

Sample	Photo	Thickness [mm]
P	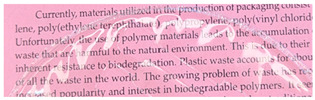	0.080 ± 0.003
PV1	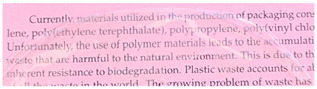	0.081 ± 0.002
PV2	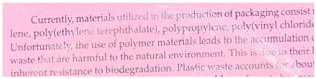	0.079 ± 0.003
PV3	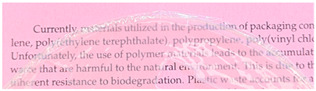	0.082 ± 0.002

**Table 2:** DSC data for the studied materials.

Sample	T_g_ (°C)	T_c_ (°C)	ΔH_c_ (J/g)	T_m_ (°C)	ΔH_m_ (J/g)	X_c_ [%]
P	44.69	105.43	5.80	144.93/152.01	6.04	5.83
PV1	41.52	99.29	3.87	144.84/152.05	4.85	4.73
PV2	40.77	104.04	3.30	144.47/150.55	4.49	4.47
PV3	38.40	113.12	3.23	150.89	4.35	4.29

## Data Availability

The original contributions presented in the study are included in the article; further inquiries can be directed to the corresponding author.
